# Risk factors of transient tachypnea of the newborn developing into pulmonary hypertension of the newborn: a case-control study

**DOI:** 10.2478/abm-2022-0034

**Published:** 2023-08-01

**Authors:** Gunlawadee Maneenil, Waricha Janjindamai, Supaporn Dissaneevate, Anucha Thatrimontrichai

**Affiliations:** Division of Neonatology, Department of Pediatrics, Faculty of Medicine, Prince of Songkla University, Songkhla 90110, Thailand

**Keywords:** case control, infant, newborn, pulmonary hypertension, risk factors, transient tachypnea of the newborn

## Abstract

**Background:**

Transient tachypnea of the newborn (TTN) is the consequence of delayed resorption of lung fluid. When TTN develops, the infant may develop severe hypoxemia and progress to persistent pulmonary hypertension of the newborn (PPHN).

**Objectives:**

To examine factors associated with the development of PPHN in TTN infants.

**Methods:**

This retrospective study comprised 23 infants in whom a diagnosis of TTN with PPHN (TTN-PPHN) was confirmed and 59 infants with severe TTN without PPHN who required mechanical ventilation between 2009 and 2018 at Songklanagarind Hospital, Thailand. Logistic regression was used to assess factors associated with TTN and PPHN.

**Results:**

The factors identified by univariate analysis that were associated with development of PPHN were oxygen saturation (SpO_2_) <90% and respiratory rate (RR) ≥70 breaths/min at the time of admission, mean airway pressure (MAP) ≥8 cmH_2_O, oxygen index (OI) ≥10, partial pressure of oxygen (PaO_2_) ≤60, partial pressure of carbon dioxide (PCO_2_) ≥45 mmHg, and infants who did not receive positive pressure ventilation (PPV). In multivariate analyses, RR ≥70 breaths/min (adjusted odds ratio [aOR] 9.96, 95% confidence interval [CI] 2.1–47.29, *P* < 0.001) and OI ≥10 (aOR 29.22, 95% CI 4.46–191.23, *P* < 0.001) remained statistically significantly associated with PPHN.

**Conclusions:**

High RR and high OI were factors associated with PPHN in TTN infants.

Transient tachypnea of the newborn (TTN) is a common cause of respiratory distress in newborns, and is believed to result from incomplete clearance of lung fluid. TTN is a self-limited disease and the symptoms generally resolve within 72 h of age [1]. However, some infants with TTN may develop severe hypoxemia and persistent pulmonary hypertension of the newborn (PPHN) [2]. Pulmonary hypertension in these infants can occur due to elevation of pulmonary vascular resistance (PVR) from absorption atelectasis and the formation of reactive oxygen species [3]. Previous studies reported that meconium aspiration syndrome, asphyxia, sepsis, and maternal diabetes mellitus were associated with an elevated risk for PPHN [4, 5]. One study reported that some of their TTN infants had refractory pulmonary hypertension, and required intensive care and extracorporeal membrane oxygenation (ECMO) therapy [6]. These infants are at risk of severe complications such as neurodevelopmental impairment, or hearing impairment [5]. Therefore, the aims of this study were to examine factors associated with the development of PPHN in TTN infants and the neonatal outcomes including ventilator-associated pneumonia (VAP), ventilator days, and length of hospital stay (LOS).

## Methods

The study was approved by the Human Research Ethics Committee Faculty of Medicine, Prince of Songkla University, Thailand (Certificate of approval No. REC 60-387-01-1).

### Study population

We conducted a retrospective study of 82 infants diagnosed with TTN between January 2009 and June 2018 at the neonatal intensive care unit (NICU) of Songklanagarind Hospital. The NICU is a level III in a university hospital, which is the referral center in Southern Thailand. The infants were divided into 2 groups: 23 infants diagnosed with TTN with PPHN (TTN-PPHN) and 59 infants with severe TTN. The diagnosis of TTN was based on an infant's clinical presentation within 24 h after birth, physical examination findings, classic chest radiographic findings, and the exclusion of all other known respiratory disorders [1, 2, 3, 4, 5, 6]. Severe TTN was defined as a TTN infant who required endotracheal intubation and mechanical ventilation but without PPHN. PPHN was suspected based on a history of cyanosis, labile hypoxemia, and differential cyanosis (preductal and postductal oxygen saturation (SpO_2_) difference more than 5%–10%) [2]. All PPHN diagnoses were confirmed by echocardiography and showed evidence of increased pulmonary pressure and the presence of a right-to-left shunt at ductus arteriosus or foramen ovale. We excluded TTN infants who had mild symptoms or did not require invasive mechanical ventilation. The other exclusion criteria were the presence of a life-threatening congenital anomaly, chromosomal abnormality, and congenital heart disease.

All of the patients in this study were managed according to our NICU protocols, which included mechanical ventilation adjusted by the attending physicians. The inotropic agents were adjusted to maintain mean arterial pressure or systolic blood pressure (SBP) at the 50th percentile for gestation age.

### Data collection

Data on gestational age (GA), birth weight, sex, delivery mode, Apgar scores, and intervention at the delivery room were collected. Positive pressure ventilation (PPV) was performed with T-piece resuscitator when necessary. The initial peak inspiratory pressure (PIP) and positive end-expiratory pressure (PEEP) were 20 cmH_2_O and 5 cmH_2_O, respectively. Respiratory parameters at the time of admission, such as respiratory rate (RR), SpO_2_, ventilator setting, and umbilical arterial blood gas or arterial blood gas after intubation, were also collected. We also determined oxygen index (OI = FiO_2_ × mean airway pressure [MAP] × 100/partial pressure of oxygen [PaO_2_]), which is the parameter to assess oxygenation in infants with hypoxic respiratory failure [7]. The first OI value was calculated after the patient was put on mechanical ventilation for 30 min. Maternal and obstetric data were collected, i.e., maternal age, underlying diseases (asthma, gestational diabetes, pregnancy-induced hypertension [PIH]), mode of delivery, and obstetric complications (placenta previa, postpartum hemorrhage). The recorded GA was based on the last menstrual period or early prenatal ultrasound examination findings.

Treatment outcome data collected included the number of inotropic agents used, pneumothorax, oxygen supplementation duration, ventilator days, LOS, overall hospital cost, and neonatal screening that included hearing screening (otoacoustic emission technique) and cranial ultrasound results. Cranial ultrasonography was performed by a pediatric radiologist and the findings were classified into 4 grades of severity [8]. Grading of intraventricular hemorrhage (IVH) was carried out as follows: grade 1, germinal matrix hemorrhage <10% IVH; grade 2, IVH 10%–50%; grade 3, IVH >50%, usually with distention of lateral ventricle; and grade 4, periventricular echo-density signifying parenchymal lesion.

### Statistical analysis

The Epicalc package of the R program version 3.3.1 (R Foundation for Statistical Computing) was used for the statistical analysis. The Shapiro–Wilk normality test was used to determine whether the sample values were suited to a normal distribution or not. Descriptive statistics for continuous variables are presented as mean ± standard deviation (SD) and median (interquartile range [IQR]). Nominal variables are expressed as numbers and percentages. The Wilcoxon rank-sum test and Fisher exact test were used to compare differences between the TTN-PPHN and severe TTN groups. We performed univariate and multivariate analyses to assess the factors associated with TTN and PPHN. Variables with *P* < 0.2 in the univariate analysis were entered into backward stepwise logistic regression models in the multivariate analysis. Statistical significance was set at a *P* < 0.05.

## Results

During the study period, 883 infants were diagnosed with TTN. Of these infants, 82 with severe clinical conditions requiring endotracheal intubation and mechanical ventilation were enrolled in the study. Out of the infants with TTN, 23 were diagnosed with PPHN (TTN-PPHN); and 59 infants were diagnosed with severe TTN without PPHN. The mean ± SD GA and birth weight of all infants were 36.1 ± 2.1 weeks and 2712 ± 675 g, respectively. Fifty-six percent of the infants (46/82) were male sex and 89% of the infants (73/82) were delivered by cesarean section. The medians (IQR) of onset of respiratory distress and age at intubation were 0 (0–1) h and 2.8 (1–8) h, respectively. The medians (IQR) of RR and SpO_2_ at the time of admission in all infants were 68 (64–78) breaths/min and 90% (88%–95%), respectively.

**Table 1** shows the comparisons of the baseline demographics and clinical characteristics between the infants diagnosed with TTN-PPHN and severe TTN. The infants diagnosed with TTN-PPHN had significantly higher RR, MAP, and OI as well as lower SpO_2_, which were significantly different from those of the severe TTN infants. The severe TTN infants received significantly higher PPV in the delivery room than the TTN-PPHN infants (33.9% vs. 8.7%, *P* = 0.04). However, the numbers of infants who received early continuous positive pressure were not significantly different between the 2 groups (TTN-PPHN 21.7% vs. severe TTN 32.2%, *P* = 0.51). The neonatal characteristics of gender, GA, birth weight, Apgar scores, blood sugar, and hemoglobin level were not significantly different between the 2 groups. In addition, the maternal factors, which included mode of delivery, underlying diseases, and obstetric complications, were not significantly different between the 2 groups.

**Table 1.** Baseline demographic and clinical characteristics

	**TTN-PPHN (n = 23)**	**Severe TTN (n = 59)**
**Maternal factors**		
Cesarean section	22 (95.7)	51 (86.4)
Gestational diabetes	3 (13)	4 (6.8)
Maternal asthma	1 (4.3)	1 (1.7)
PIH	0 (0)	9 (15.3)
**Neonatal factors**		
GA (weeks)	36.5 ± 1.5	36 ± 2.3
Male	12 (52.2)	34 (57.6)
Birth weight (g)	2825 ± 517	2668 ± 727
Apgar score at 1 min	8 (8–8.5)	8 (6–8)
Apgar score at 5 min	9 (9–9)	9 (8–9)
PPV	2 (8.7)	20 (33.9)
Creatinine – 24 h (mg/dL)	0.6 ± 0.1	0.7 ± 0.2
Creatinine – 48 h (mg/dL)	0.7 ± 0.2	0.7 ± 0.2
**Respiratory parameters at the time of admission**		
RR (breaths/min)	72 (69–80)	66 (63–73)
SpO_2_ (%)	88 (85–91.5)	90 (89–95)
MAP (cmH_2_O)	8 (7.7–9)	7 (7–8)
OI	6.5 (4.2–14.3)	3.1 (2.1–5.1)
**Arterial blood gas results**		
pH	7.3 (7.2–7.3)	7.3 (7.3–7.4)
PCO_2_	42.9 ± 10.5	38.1 ± 10.2
PaO_2_	62 (50.5–115)	93 (65–132)

The results are expressed as n (%), mean ± SD, or median (IQR).

GA, gestational age; IQR, interquartile range; MAP, mean airway pressure; OI, oxygen index; PaO_2_, partial pressure of oxygen; PCO_2_, partial pressure of carbon dioxide; PIH, pregnancy-induced hypertension; PPHN, persistent pulmonary hypertension of the newborn; PPHN, persistent pulmonary hypertension of the newborn; PPV, positive pressure ventilation; RR, respiratory rate; SD, standard deviation; SpO_2_, oxygen saturation; TTN, transient tachypnea of the newborn.

The univariate and multivariate analyses of the risk factors between the TTN-PPHN and severe TTN infants are summarized in **Table 2**. Using univariate analysis, the following were the factors ascertained to be associated with the development of PPHN, not receiving PPV after birth, or who had RR ≥70 breaths/min, SpO_2_ ≤90%, MAP ≥8 cmH_2_O, OI ≥10, PaO_2_ ≤60 mmHg and partial pressure of carbon dioxide (PCO_2_) ≥45 mmHg. Using multivariate regression analysis, it was then found that RR ≥70 breaths/min at the time of admission (*P* < 0.001) and OI ≥10 (*P* < 0.001) were associated with PPHN.

**Table 2.** Univariate and multivariate analyses of the factors between infants diagnosed with TTN-PPHN (n = 23) and infants with severe TTN (n = 59)

**Variable**	**Univariate analysis**			**Multivariate analysis**		

	**Crude OR**	**95% CI**	***P***	**Adjusted OR**	**95% CI**	***P***
Received PPV, no vs. yes	5.38	1.15–25.29	0.01	9.96	2.1–47.29	<0.001
RR ≥70 breaths/min	4.43	1.52–12.9	0.004			
SpO_2_≤90%	3.21	1.18–8.72	0.02			
MAP ≥8 cmH_2_O	3.90	1.34–11.35	0.009	29.22	4.46–191.23	<0.001
OI ≥10	11.57	2.76–48.49	<0.001			
PaO_2_≤60 mmHg	5.61	1.85–17.0	0.002			
PCO_2_≥45 mmHg	3.06	1.12–8.32	0.03			

CI, confidence interval; MAP, mean airway pressure; OI, oxygen index; PaO_2_, partial pressure of oxygen; PCO_2_, partial pressure of carbon dioxide; PPHN, persistent pulmonary hypertension of the newborn; PPV, positive pressure ventilation; RR, respiratory rate; SpO_2_, oxygen saturation; TTN, transient tachypnea of the newborn.

The median (IQR) age at the onset of PPHN and the median (IQR) initial OI in the TTN-PPHN infants were 17 (10–34.5) h and 6.5 (4.2–14.3), respectively. Of the 23 infants with TTN-PPHN, 52% (12/23) received inhaled nitric oxide (iNO) therapy. The initial dose of iNO was 20 ppm and the mean ± SD duration of iNO was 76.3 ± 31 h. Pulmonary vasodilators were prescribed in cases of mild to moderate PPHN (OI <20) or used as an adjunct therapy to iNO. Among the infants, 4 received sildenafil and 15 received bosentan therapy. The mean ± SD durations of sildenafil and bosentan therapies were 7 ± 6.2 d and 6.6 ± 2.5 d, respectively.

We conducted assessments for acute kidney injury (AKI), which is a common comorbidity of critically ill neonates. None of the infants had AKI according to the neonatal KDIGO classification [9]. There was a statistically significant difference in serum creatinine on the first day of life between the TTN-PPHN and severe TTN infants (0.6 ± 0.1 vs. 0.7 ± 0.2, *P* = 0.03). However, serum creatinine on the second day of life and urine output were not significantly different between the 2 groups.

The neonatal outcomes are summarized in **Table 3**. The TTN-PPHN infants had a significantly higher rate of morbidities including pneumothorax and VAP than the severe TTN infants. Mechanical ventilator days, duration of oxygen supplementation, and LOS were also significantly longer in the TTN-PPHN group than in the severe TTN group. The overall hospital costs of the TTN-PPHN infants were nearly 2 times greater than the hospital costs of severe TTN infants. All infants had a normal hearing screening at discharge. Of the 23 infants (14 severe TTN infants and 9 TTN-PPHN infants) who had cranial ultrasonography performed, 2 of the severe TTN infants and 4 of the TTN-PPHN infants had IVH grade 1. There were no infant deaths on record in the data utilized in the present research.

**Table 3.** Comparison of neontal outcomes between infants diagnosed with TTN-PPHN and infants with severe TTN

**Outcomes**	**TTN-PPHN (n = 23)**	**Severe TTN (n = 59)**	***P***
Pneumothorax	4 (17.4)	2 (3.4)	0.04
VAP	5 (21.7)	2 (3.4)	0.01
Inotropic 2 agents	21 (91.3)	12 (20.3)	<0.001
Ventilator days	7 (5–11.5)	2 (1–3)	<0.001
Oxygen days	10 (9–16)	3 (2–5.5)	<0.001
LOS (d)	15 (12.2–18.8)	8 (6–11)	<0.001
Hospital cost (US$)	311.4 ±97.9	160.3 ±57.5	<0.001

The results are expressed as n (%), mean ± SD, or median (IQR).

IQR, interquartile range; LOS, length of hospital stay; PPHN, persistent pulmonary hypertension of the newborn; SD, standard deviation; TTN, transient tachypnea of the newborn; VAP, ventilator-associated pneumonia.

## Discussion

This was a retrospective study performed to examine the factors associated with the development of PPHN in TTN infants. We compared both maternal and neonatal characteristics and respiratory parameters at the time of admission in TTN infants who developed PPHN and severe TTN infants without PPHN. We found that RR at time of admission more than 70 breaths/min and OI after intubation more than 10 were the only factors associated an increased risk of developing PPHN in TTN infants. These parameters may help to predict the progression of the disease.

OI is the parameter used to assess the severity of hypoxic respiratory failure. An OI >25 is the criterion for iNO therapy while OI >40 is the criterion for ECMO therapy, which is associated with a higher mortality [10]. González et al. [11] reported that the early use of iNO in infants with moderate respiratory failure (OI 10–30) would improve oxygenation and decrease the probability of developing severe hypoxemic respiratory failure (OI >40). Kumar et al. [12] reported on outcomes by the highest OI in the first 24 h of admission in neonates with hypoxemic respiratory failure and found that no infants with OI <14 died while 80% of the total deaths had an OI >25.

Kahvecioğlu et al. [13] reported on clinical clues to predict the severity of disease and need for respiratory support in TTN infants. They found that the severe TTN infants had higher Silverman and Richardson scores than the mild TTN infants [14]; however, RRs were not significantly different between the 2 groups. Kasap et al. [15] examined the factors associated with prolonged tachypnea in TTN infants and found that a peak RR more than 90 breaths/min during the first 36 h was associated with an increased risk of prolonged tachypnea. Therefore, they suggested that peak RR could help to predict the duration of tachypnea. Besides the clinical tools, a previous study reported the role of plasma N-terminal pro-B-type natriuretic peptides (NT-proBNP) in predicting infants with TTN who would have prolonged tachypnea and require mechanical ventilation [16].

It is known that TTN is associated with PPHN. One of the most important factors related to both TTN and PPHN is cesarean section [17], which was also noted in our study. The rate of cesarean section in all infants in this study was 89% (73/82). Of these 73 infants, 21.9% (16/73) were elective cesarean section (ECS) and the mean ± SD GA of all infants was 36.1 ± 2.1 weeks. In our hospital, 3500 deliveries are performed annually and nearly 50% of these are cesarean section. The decision for ECS depends on the opinion of the obstetrician. Recent studies have reported that ECS at 37–38 weeks had a higher risk of neonatal morbidity such as respiratory distress, asphyxia, the need for intubation or continuous positive airway pressure (CPAP), and mortality [18]. We suggest that scheduling elective cesarean delivery at 39 weeks gestation is the optimal timing with the potential benefit of reducing the risk of developing TTN and PPHN, which follows the ACOG recommendations.

In the univariate analysis, we found that infants who did not receive PPV had increased risk of developing PPHN (crude OR 5.38, 95% confidence interval [CI] 1.15–25.29, *P* = 0.01), while the use of early CPAP in the delivery room was not associated with PPHN. On the other hand, recent publications have reported that the use of early CPAP in TTN infants reduced the duration and severity of respiratory distress and decreased the rate of NICU admission [19, 20].

A previous study reported that factors associated with an elevated risk for PPHN included cesarean delivery, diabetes, and asthma [21]. In contrast, because of our small sample size, we did not find any maternal factors that were associated with PPHN. We also did not find the neonatal factors such as GA <37 weeks or large or small for GA, which were the factors associated with PPHN as in another previous study [22].

PPHN is associated with significant morbidity and mortality. The outcomes of the infants in our study with TTN-PPHN included VAP, pneumothorax, ventilator days, and overall hospital costs, all of which was significantly different from the outcomes of the severe TTN infants. There were no infant deaths on record in the data comprising the study, which indicated that TTN was not an aggressive disease.

Our study had several limitations. This was a small retrospective study, which limited the study power. We enrolled only TTN infants who required endotracheal intubation and mechanical ventilation, which also possibly affected the study power. Furthermore, there was a lack of long-term follow-up visits in these infants for neurodevelopmental outcomes evaluation.

In conclusion, we found that RR ≥70 breaths/min at the time of admission and OI ≥10 were the risk factors for developing PPHN in TTN infants. Clinicians should provide intensive care, monitor preductal and postductal SpO_2_, and perhaps perform echocardiography if indicated for an early diagnosis of PPHN in these infants.
